# The metabolic cost of maintaining a synapse during development

**DOI:** 10.1186/1471-2202-14-S1-P203

**Published:** 2013-07-08

**Authors:** Jan Karbowski

**Affiliations:** 1 Institute of Biocybernetics and Biomedical Engineering, Polish Academy of Sciences, Warsaw, Poland; 2 Institute of Applied Mathematics and Mechanics, University of Warsaw, Warsaw, Poland

## Background

Experimental data indicate that cerebral energy consumption and synaptic density qualitatively correlate during development [1,2], presumably indicating that energy plays a key role during the process. The purpose of this study is two-fold. First, to collect empirical data on cortical metabolism and synaptic density during development (from birth to adulthood for different mammals), and to quantify their dependence. Second, to provide atheoretical link between neural metabolism and electrical activities of neurons and synapses [3]. A theoretical model allows us to estimate synaptic contribution to the overall energy used by neurons.

## Results

It is found that regional metabolic rate per synapse is approximately conserved from birth to adulthood for a given species (rat, cat, macaque, human). A typical synapse uses about 7000 glucose molecules per second in primate cerebral cortex, and about 5 times of that amount in cat and rat visual cortices [4]. Based on the theoretical model it is found that synaptic efficacy is generally inversely correlated with average firing rate, and additionally, synapses consume a bulk of metabolic energy, roughly 50-90 % during most of the developmental process (except human temporal cortex < 50 %) [4]. Overall, these results suggest a tight regulation of brain electrical and chemical activities during the formation and consolidation of neural connections. This presumably reflects strong energetic constraints on brain development [4].
